# GNE deficiency impairs Myogenesis in C2C12 cells and cannot be rescued by ManNAc supplementation

**DOI:** 10.1093/glycob/cwae004

**Published:** 2024-01-15

**Authors:** Carolin T Neu, Linus Weilepp, Kaya Bork, Astrid Gesper, Rüdiger Horstkorte

**Affiliations:** Institute for Physiological Chemistry, Medical Faculty, Martin Luther University Halle-Wittenberg, 06114 Halle (Saale), Germany; Institute for Physiological Chemistry, Medical Faculty, Martin Luther University Halle-Wittenberg, 06114 Halle (Saale), Germany; Institute for Physiological Chemistry, Medical Faculty, Martin Luther University Halle-Wittenberg, 06114 Halle (Saale), Germany; Institute for Physiological Chemistry, Medical Faculty, Martin Luther University Halle-Wittenberg, 06114 Halle (Saale), Germany; Institute for Physiological Chemistry, Medical Faculty, Martin Luther University Halle-Wittenberg, 06114 Halle (Saale), Germany

**Keywords:** GNE, GNE myopathy, Polysialylation, Posttranslational modification, Sialic acid biosynthesis

## Abstract

GNE myopathy (GNEM) is a late-onset muscle atrophy, caused by mutations in the gene for the key enzyme of sialic acid biosynthesis, UDP-*N*-acetylglucosamine 2-epimerase/*N*-acetylmannosamine kinase (GNE). With an incidence of one to nine cases per million it is an ultra-rare, so far untreatable, autosomal recessive disease. Several attempts have been made to treat GNEM patients by oral supplementation with sialic acid precursors (e.g. *N*-acetylmannosamine, ManNAc) to restore sarcolemmal sialylation and muscle strength. In most studies, however, no significant improvement was observed. The lack of a suitable mouse model makes it difficult to understand the exact pathomechanism of GNEM and many years of research have failed to identify the role of GNE in skeletal muscle due to the lack of appropriate tools. We established a CRISPR/Cas9-mediated *Gne*-knockout cell line using murine C2C12 cells to gain insight into the actual role of the GNE enzyme and sialylation in a muscular context. The main aspect of this study was to evaluate the therapeutic potential of ManNAc and *N*-acetylneuraminic acid (Neu5Ac). Treatment of *Gne*-deficient C2C12 cells with Neu5Ac, but not with ManNAc, showed a restoration of the sialylation level back to wild type levels–albeit only with long-term treatment, which could explain the rather low therapeutic potential. We furthermore highlight the importance of sialic acids on myogenesis, for C2C12 *Gne*-knockout myoblasts lack the ability to differentiate into mature myotubes.

## Introduction

**Fig. 1:**
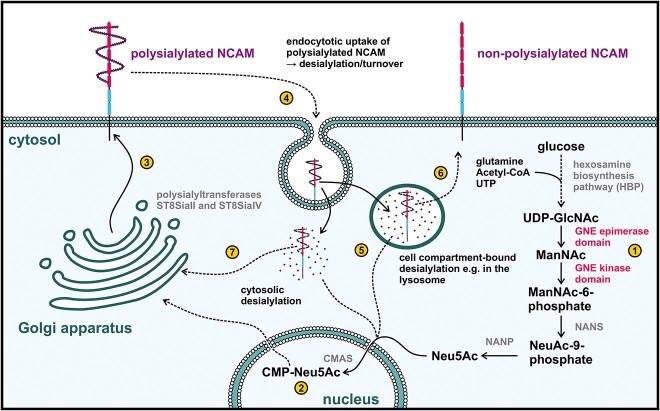
**Schematic drawing of the sialic acid biosynthesis and degradation pathway.** (1) UDP-lcNAc, derived from the hexosamine biosynthetic pathway, is the substrate for the epimerase domain of GNE, initiating sialic acid biosynthesis. The resulting ManNAc is subsequently phosphorylated by the GNE-kinase domain. Enzymatic activity of NANS and NANP lead to formation of Neu5Ac, which is then translocated into the nucleus by an, yet unknown, mechanism. (2) nucleotide-activation of Neu5Ac follows via CMAS and CMP-Neu5Ac can finally enter, though the cytoplasm, into the Golgi apparatus, where membrane-resident sialyltransferases (ST8SiaII and ST8SiaIV) mediate the transfer of sialic acids to the termini of nascent glycans (3 and 4) for desialylation, polysialylated NCAM is endocytosed into the cell (Monzo et al. 2013), and desialylated in the cytosol or in a cellular compartment–Most likely the lysosome–Providing an additional source of sialic acids for the cell (5). Further investigations are needed to elucidate the exact desialylation pathway of NCAM. NCAM can then return to the membrane–Now in a non-polysialylated form (6) or it can be re-polysialylated in the Golgi apparatus (7).

Sialic acids are a subset of α-keto acid monosaccharides, with *N*-acetylneuraminic acid (Neu5Ac) being the most abundant one in humans (Varki 2008). In mammalian cells, the first two steps of sialic acid biosynthesis are catalysed by the bifunctional enzyme UDP-*N*-acetylglucosamine 2-epimerase/*N*-acetylmannosamine kinase (GNE). The N-terminal epimerase domain of GNE uses UDP-*N*-acetylglucosamine (UDP-GlcNAc) as a substrate and epimerises it into *N*-acetylmannosamine (ManNAc), which is subsequently phosphorylated by the C-terminal kinase domain (Hinderlich et al. 1997) (Fig. 1). Three more enzymes, *N*-acetylneuraminate synthase (NANS), *N*-acetylneuraminic acid phosphatase (NANP), and cytidine monophosphate *N*-acetylneuraminic acid synthetase (CMAS), are needed to finalize sialic acid synthesis in the cytosol and its activation in the nucleus, respectively (Roseman et al. 1961; Munster-Kuhnel et al. 2004; Maliekal et al. 2006). Activated CMP-Neu5Ac passes from the nucleus to the Golgi apparatus, where membrane-resident sialyltransferases transfer sialic acids to nascent glycoproteins *en route* to the plasma membrane and extracellular space. Sialic acids decorate the terminal ends of glycan structures, modulating protein characteristics like half-life and solubility, as well as cellular mechanisms like migration, adherence, and immune signalling (Bhide and Colley 2017).

It has been found that bi-allelic mutations in the *GNE* gene can cause a rare genetic condition called GNE myopathy (GNEM, OMIM 605820), formerly also known as hereditary inclusion body myopathy (HIBM) (Eisenberg et al. 2001). Other disorders caused by mutations in the *GNE* include sialuria (OMIM 269921) (Seppala et al. 1999) and thrombocytopenia (Revel-Vilk et al. 2018).

GNEM patients suffer from muscle weakness and atrophy with a slow progression from distal to proximal beginning in the lower limbs but excluding the quadriceps (Pogoryelova et al. 2018). Manifestation of the disease usually starts during early adulthood, and patients are mostly wheelchair dependent 10 to 15 years after the onset of the disease (Pogoryelova et al. 2018). Pathohistological findings show characteristic rimmed vacuoles containing protein aggregates, among which amyloid β and hyperphosphorylated tau have been identified (Nonaka et al. 1981; Argov and Yarom 1984; Carrillo et al. 2018; Devi et al. 2018). Although not a criterion for GNEM, inflammation occurs in some patients, especially in the early stages (Krause et al. 2003; Yabe et al. 2003).

Since GNE is known for its role in sialic acid biosynthesis, it is tempting to assume that impaired GNE activity leads to hyposialylation in skeletal muscle tissue. Indeed, dynamic remodelling of sarcolemmal glycan structures has been observed during myogenesis, suggesting stringent regulation of sialic acid content for proper muscle function (Hinderlich et al. 1997; Blazev et al. 2021).

Most interestingly, a recent study on a patient-derived iPSC model of GNEM shows myogenic defects compared to control cells (Schmitt et al. 2022). Myogenic impairment was also observed in a cell model, using the murine Sol8 skeletal muscle cell line (Ilouz et al. 2022). These findings are further supported by the finding, that GNE plays an important role for skeletal muscle differentiation during embryonic differentiation in mice (Milman et al. 2011).

To prevent hyposialylation, the development of supplementation therapies with sialic acid metabolites such as ManNAc is currently the preferred approach for GNEM patients. A first in-human trial of oral ManNAc administration significantly increased the sialic acid levels in the patients’ plasma (Xu et al. 2017). A current review of clinical trials to treat GNEM patients can be found here (Yoshioka et al. 2022). However, all studies have failed so far to significantly ameliorate the patient’s outcome, leaving the question on how these attempts may be improved.

In the present study, we established a *Gne*-knockout cell model, using the murine myoblast cell line C2C12 to study the efficiency of exogenous ManNAc and sialic acid (Neu5Ac) administration in a skeletal muscular background. First, we found that the absence of GNE prevents the differentiation of myoblasts into mature myotubes, as described previously in other studies (Ilouz et al. 2022; Schmitt et al. 2022). Noteworthy, an excess of Neu5Ac during the course of differentiation impedes myogenesis in C2C12 wild type cells. Furthermore, supplementation of C2C12 *Gne*-knockout cells with ManNAc did not show any restoration of polysialylation as was previously reported for other models (Hinderlich et al. 2001; Malicdan et al. 2009; Niethamer et al. 2012). These results may help explain why clinical trials in patients have failed so far and suggest re-thinking new therapeutic approaches (Lochmuller et al. 2019).

## Results

### Supplementation of GNE-deficient HEK-293 cells with the Neu5Ac-precursor ManNAc

The therapeutic approaches to treat GNEM with *N*-acetylmannosamine (ManNAc) are based on experiments in GNE-deficient cells and in a GNEM mouse model, which showed increased sialylation after supplementation with ManNAc (Hinderlich et al. 2001; Malicdan et al. 2009; Peters et al. 2023).

**Fig. 2:**
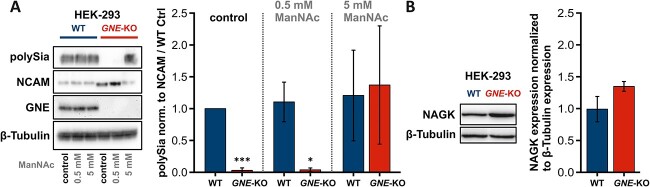
**Supplementation of HEK-293 GNE-knockout cells with ManNAc**. A) Representative western blots showing the expression of polySia, its carrier protein NCAM, GNE, and β-tubulin in HEK-293 wild type and GNE-knockout cells in relation to supplementation with different concentrations of ManNAc. B) Representative western blots showing the expression of GlcNAc kinase (NAGK) and β-tubulin in HEK-293 wild type and GNE-knockout cells. β-tubulin is used as a loading control in (a and B). The quantification data in the bar graph represent the mean of three independent experiments (*n* = 3). The error bars represent the standard deviation. *P*-values were calculated using Student’s t-test and are given here in relation to the wild type control. Asterisks above the bars were used to classify Student’s t-test results according to different levels of significance: *P* ≤ 0.05: ^*^ and *P* ≤ 0.005:^*^^*^^*^.

Hence, as a proof of function, we treated human embryonal kidney cells (HEK-293) wild type and *GNE*-knockout cells with 0.5 mM and 5 mM ManNAc, respectively, for 24 h. A monoclonal antibody, recognizing α-2,8-linked sialic acids (polySia) was used to depict the sialylation status of the cells in the Western blot analysis (Peters et al. 2023). *GNE*-knockout cells showed no expression of polysialylation, highlighting the unique role of GNE in sialic acid biosynthesis (Fig. 2A). While treatment with 0.5 mM ManNAc had no effect on the polysialylation level of the *GNE*-knockout cells, treatment with tenfold higher doses of ManNAc (5 mM) restored polysialylation to wild type levels. In wild type cells, however, the degree of polysialylation could not be influenced, regardless of the ManNAc concentration used. One explanation for this could be the already known feedback-inhibition mechanism triggered by CMP-Neu5Ac (Seppala et al. 1991). NCAM is one of the few proteins that have been found to be polysialylated in cells (Muhlenhoff et al. 1996; Bhide and Colley 2017). We used it here as an additional control and assumed it to be the main carrier of polySia in our cell model. The lower and more intense bands in the untreated and 0.5 mM treated *GNE*-knockout cells represent the un-polysialylated form of NCAM, being better susceptible for the antibody (Fig. 2A).

These results raise the question of how ManNAc is phosphorylated for the further biosynthetic steps in the absence of the GNE kinase domain. It has been shown that the N-acetylglucosmaine kinase (NAGK) is able to phosphorylate ManNAc, and is thus believed to bypass the lack of GNE kinase activity in GNE-deficient cells (Allen and Walker 1980). A recent study also suggests that external ManNAc is mainly phosphorylated by NAGK (Gorenflos Lopez et al. 2023). Western Blot analysis showed a trend towards higher NAGK expression in *GNE*-knockout cells (*P*-value: 7.47E-02, Fig. 2B), augmenting the putative role of NAGK in ManNAc phosphorylation in vitro.

### Establishment of Gne-deficient C2C12 cells

**Fig. 3:**
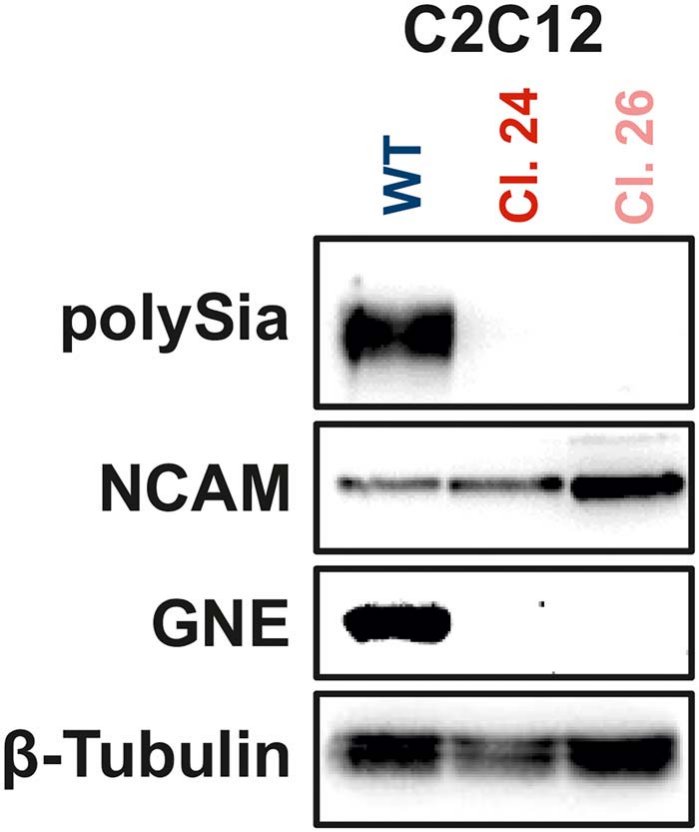
**Western blot characterization of C2C12 wild type and Gne-deficient C2C12 cells.** Representative western blots showing the expression of polySia, NCAM, GNE, and β-tubulin in C2C12 wild type and in C2C12 Gne-deficient single cell clones number 24 and 26.

GNEM is caused by bi-allelic mutations in the *GNE* gene, leading to muscle weakness and atrophy. Since it is exclusively the skeletal muscle tissue that is affected in patients, we asked whether our results from the HEK-293 model (Fig. 2) could be reproduced in the murine myoblast cell line C2C12. Using the CRISPR/Cas9 system, we transfected C2C12 wild type cells and selected single cell clones 24 and 26 as positive *Gne-*knockout clones. As proven by western blot, clones 24 and 26 lack GNE protein expression along with polySia (Fig. 3). Noteworthy, clone 26 presents a slightly different phenotype than clone 24, having a stronger NCAM expression, arising from single cell heterogeneity (Fig. 3).

**Fig. 4:**
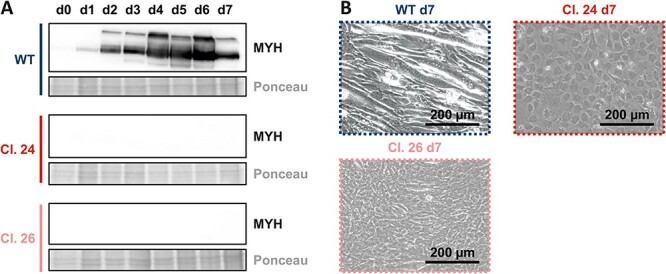
**Impaired myogenesis in C2C12 Gne-knockout cells. A)** Representative western blots showing the expression of myosin heavy chain (MYH) in C2C12 wild type and in the C2C12 Gne-knockout single cell clones 24 and 26. B) Representative microscopic images of these cells after differentiation day 7. Western blots and microscopic images each represent the results of one of three representative biological replicates.

Notably, *Gne*-knockout in mice results in embryonic lethality between E8.5 and E9.5, demonstrating the role of sialic acids during development (Schwarzkopf et al. 2002). While C2C12 wild type cells were fully differentiated into myotubes after 7 days of differentiation, this phenotype was absent in *Gne*-knockout cells. This was confirmed on molecular level by the absence of myosin heavy chain (MYH) a common marker of myogenesis (Fig. 4A). In addition, the microscopic pictures of the cells on differentiation day 7 demonstrate the highly different phenotype of both *Gne*-knockout clones compared to wild type myotubes (Fig. 4B). This seems to indicate that sialic acids play a crucial role in myotube formation.

### Supplementation of Gne-deficient C2C12 cells with ManNAc and Neu5Ac for 24 h

**Fig. 5:**
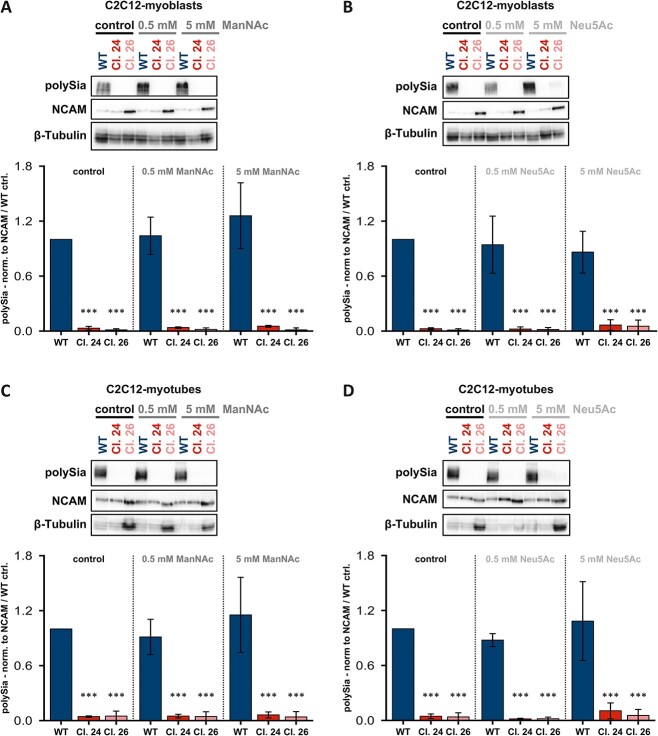
**Treatment of C2C12 cells with ManNAc and Neu5Ac for 24 h. A)** Representative western blots showing the expression of polySia, NCAM, and β-tubulin after 24 h treatment of C2C12 myoblasts with different concentrations of ManNAc. B) Representative western blots showing the expression of polySia, NCAM, and β-tubulin after 24 h treatment of C2C12 myoblasts with different concentrations of Neu5Ac. C) Representative western blots showing the expression of polySia, NCAM, and β-tubulin after 24 h treatment of C2C12 myotubes with different concentrations of ManNAc. Note that the term “Myotube” refers to the way the cells were cultured and WT morphology, respectively. Gne knock out clones don’t fuse into myotubes under these experimental conditions. D) Representative western blots showing the expression of polySia, NCAM, and β-tubulin after 24 h treatment of C2C12 myotubes with different concentrations of Neu5Ac. The bar graphs below the western blots in each subpanel show the polySia level normalized to the NCAM level in the C2C12 wild type control. The quantification data in the bar graph represent the mean of three independent experiments (*n* = 3). The error bars represent the standard deviation. *P*-values were calculated using Student’s t-test and are given here in relation to the untreated wild type control. Asterisks above the bars were used to classify Student’s t-test results according to different levels of significance: *P* ≤ 0.005:^*^^*^^*^.

Since we were able to restore polysialylation in *GNE*-deficient HEK-293 cells, we next checked whether this also works in our *Gne*-knockout skeletal muscle cell line. To do this, we treated C2C12 *Gne*-knockout myoblasts with ManNAc for 24 h. Surprisingly, ManNAc exhibited no effect on these myoblasts, as there was no polySia detectable by Western blot, even at high concentrations of 5 mM (Fig. 5A). To enhance ManNAc uptake into cells, we also treated C2C12 *Gne*-knockout cells with peracetylated ManNAc (Ac_4_ManNAc), which exhibits enhanced membrane permeability. However, even supplementation with Ac_4_ManNAc did not show any positive effects in terms of a desired restoration of polysialylation (Suppl. Fig. 1).

In another experiment, since we do not know whether the lack of polysialylation recovery is due to poor uptake of ManNAc into muscle cells or rather to the absence of key enzymes, we treated the cells with sialic acids (Neu5Ac) for 24 h. As with ManNAc treatment, Neu5Ac failed to restore polysialylation levels in clones 24 and 26 (Fig. 5B). These findings also apply to the treatment of differentiated myotubes. ManNAc (Fig. 5C) and Neu5Ac (Fig. 5D) had no effect on the degree of polysialylation in either wild type or *Gne*-knockout cells. Please note that the term “myotube” refers to the way the cells were cultured and WT morphology, respectively. Gne knock out clones don’t fuse into myotubes under these experimental conditions.

To rule out cell line specific effects, we repeated the experiments in another murine myoblast cell line, Sol8. The results already observed in C2C12 *Gne*-knockout cells could be confirmed in the Sol8 cell line and showed no recovery/or improvement of the degree of polysialylation there either (Suppl. Fig. 2).

**Fig. 6:**
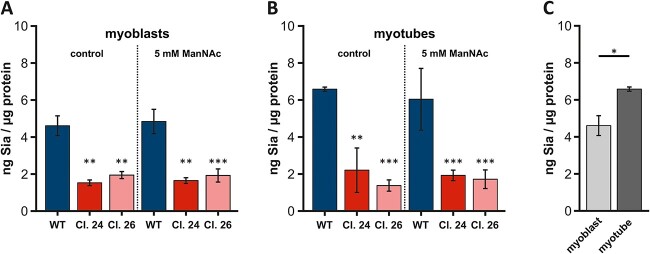
**Sialic acid quantification via resorcinol assay**. A) Comparison of sialic acid levels in untreated and in C2C12 myoblasts (wild type and both Gne-knockout clones) treated with 5 mM ManNAc for 24 h. B) Comparison of sialic acid levels in untreated and in C2C12 myotubes (wild type and both Gne-knockout clones) treated with 5 mM ManNAc for 24 h. Note that the term “myotube” refers to the way the cells were cultured and WT morphology, respectively. Gne knock out clones don’t fuse into myotubes under these experimental conditions. C) Comparison of sialic acid content in C2C12 wild type myoblasts and myotubes. The quantification data in the bar graph represent the mean of three independent experiments (*n* = 3). The error bars represent the standard deviation. *P*-values were calculated using Student’s t-test and are given here in relation to the wild type control. Asterisks above the bars were used to classify Student’s t-test results according to different levels of significance: *P* ≤ 0.05:^*^, 0.005 < *P* ≤ 0.01:^*^^*^, *P* ≤ 0.005:^*^^*^^*^.

Since polysialylation is only one form of sialylation, which also only occurs with very few substrates, we also examined the total amount of sialic acids using the resorcinol/periodate assay. However, in accordance with the western blots of polysialylation, no increased concentration of sialic acids was observed in either myoblasts (Fig. 6A) or myotubes (Fig. 6B). Compared to the sialic acid levels of the wild type cells, the *Gne*-knockout clones 24 and 26 showed significantly lower sialic acid levels; of course, small amounts may always be endocytosed from the culture medium. However, this assumption also applies to the wild type cells and should therefore not have a major impact on the results–assuming, that the plasma membrane itself has retained the same permeability.

Furthermore, significantly more sialic acids were detected in C2C12 myotubes than in myoblasts, suggesting a dynamic remodelling of sarcolemmal glycan structure during differentiation (Fig. 6C).

To clarify why supplementation of the C2C12 muscle cell line with ManNAc did not raise sialic acid levels to wild type levels–as previously shown in HEK-293 cells (Fig. 2A), we next checked the NAGK mRNA expression. mRNA levels were significantly upregulated in clone 24 compared to wild type, in clone 26 they were at the same level as in wild type cells (Suppl. Fig. 3A).

We also checked the mRNA expression levels of the polysialyltransferases ST8SiaII and ST8SiaIV, which are known to link sialic acids in an α-2,8-conformation and thus generate long polySia chains. Our results indicate that *St8sia2* and *St8sia4* expression is downregulated in both clones in the absence of substrate availability (Suppl. Fig. 3B).

### Supplementation of Gne-deficient C2C12 cells with ManNAc and sialic acid for 8 days

The poor outcome of the 24 h treatment of C2C12 *Gne*-knockout clones with either ManNAc or Neu5Ac led to the assumption, that 24 h might be too short a period of time to have any biological relevance. Hence, we changed our experimental settings to a long-term treatment of 8 days, starting on day 0 of differentiation.

**Fig. 7:**
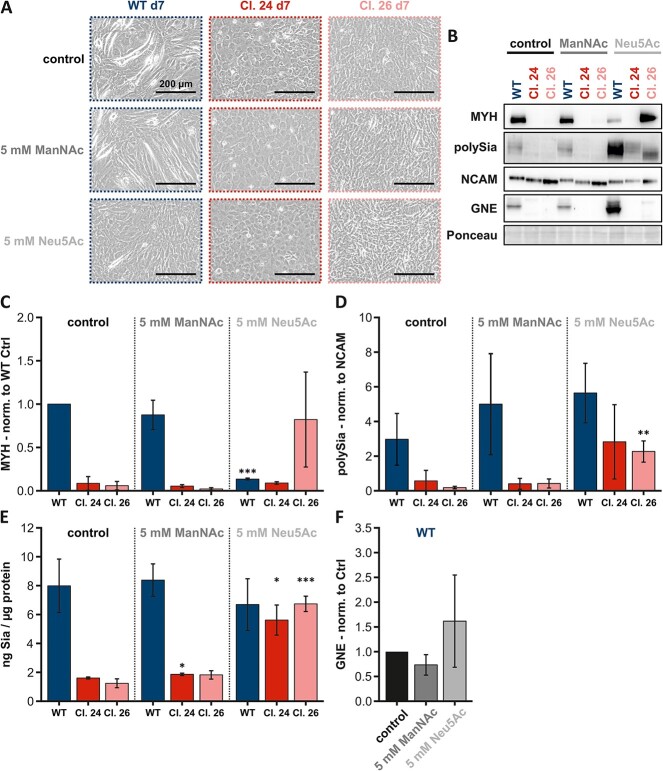
**Long-term treatment of C2C12 cells**. A)representative microscopic images of C2C12 wild type and C2C12 Gne-knockout clones 24 and 26 after differentiation day 7. B) Representative western blots showing the expression of MYH, polySia, NCAM, and GNE after treatment of the aforementioned cells with 5 mM ManNAc or 5 mM Neu5Ac for 8 days. C) Comparison of MYH expression normalized to wild type control with the aforementioned cells under the aforementioned treatment conditions. D) Comparison of the polySia level normalized to NCAM level, both under the same conditions. E) Comparison of the total amount of sialic acids per μg protein. F) Comparison of the GNE expression normalized to control. The scale bar in the microscopic images always corresponds to 200 μm. The quantification data in the bar graph represent the mean of three independent experiments (*n* = 3). The error bars represent the standard deviation. *P*-values were calculated using Student’s t-test and are given here relative to the untreated control of each cell line–C2C12 wild type and both C2C12 Gne-knockout clones. Asterisks above the bars were used to classify Student’s t-test results according to different levels of significance: *P* ≤ 0.05:^*^, 0.005 < *P* ≤ 0.01:^*^^*^, *P* ≤ 0.005:^*^^*^^*^.

Adding ManNAc to the cells during myogenesis had no effect on MYH expression (Fig. 7B, C) and cell morphology in either C2C12 wild type or the *Gne*-knockout clones (Fig. 7A). However, C2C12 wild type cells showed significantly lower MYH levels after Neu5Ac treatment (*P-*value: 2.36E-08), which matches the representative microscopic image (Fig. 7A). MYH expression was not detectable in clone 24, regardless of the substance used. In clone 26, MYH expression tended to be upregulated after treatment with Neu5Ac (*P-*value: 1.36E-01) but was also undetectable in the control and ManNAc-treated cells (Fig. 7B, C). Microscopic images of clone 24 showed no morphological differences regardless of treatment conditions and clone 26 showed very slight evidence of myotube formation after Neu5Ac treatment.

In C2C12 wild type cells, polysialylation showed a slight upward trend after either ManNAc (*P-*value: 2.77E-01) or Neu5Ac (*P-*value: 5.81E-02) treatment (Fig. 7B, D), whereas total sialic acid levels appeared unaffected by either treatment (Fig. 7E). In clone 24, polysialylation again showed a slight upward trend after Neu5Ac (*P-*value: 1.23E-01) treatment (Fig. 7B, D); the trend was confirmed by analysis of total sialic acid levels after both ManNAc (*P-*value: 1.66E-02) and Neu5Ac (*P-*value: 2.12E-02) treatment (Fig. 7E). In clone 26, polysialylation normalized to NCAM was significantly increased after Neu5Ac (*P-*value: 6.10E-03) treatment (Fig. 7B, D), again confirmed in the total sialic acid level analysis (*P-*value: 4.00E-04).

Furthermore, a trend toward upregulated GNE expression after Neu5Ac treatment was evident in the C2C12 wild type cells (Fig. 7B, F). However, the trend was not significant and needs further investigation.

Overall, it can be concluded that long-term treatment with ManNAc does not affect C2C12 cell myogenesis, the level of polysialylation, or GNE expression. Notwithstanding, in terms of restoring sialic acid content, prolonged treatment with Neu5Ac appears to be the most promising approach in GNE-deficient muscle cells.

## Discussion

GNEM is a rare genetic disease whose pathomechanism is largely unclear. A GNEM mouse model, carrying the human D207V variant was described to have a phenotype, comparable to human disease (Malicdan et al. 2007). In a follow-up study, oral treatment of these mice with sialic acid metabolites prevented muscle atrophy, proposing therapeutic applicability of sialic acid and its precursors to prevent disease progression in GNEM patients (Malicdan et al. 2009). Despite these promising results, clinical attempts to treat patients with sialic acid precursors, aiming to increase the degree of sialylation and prevent or at least slow down the progression of the disease, have failed so far. Unfortunately, the phenotype of the GNE^D207V^ mouse model was not reproducible in another study, highlighting the importance of developing further/other GNEM-disease models (Mitrani-Rosenbaum et al. 2022). Another therapeutic approach is the development and administration of ManNAc-based prodrugs that have enhanced lipophilicity and improved bioavailability (Pertusati and Morewood 2022). However, whether hyposialylation is the main cause of GNEM remains elusive. Inter-individual sialylation patterns in humans show a natural variability, hampering the distinction between healthy individuals and GNEM patients based on sialic acid levels alone (Sela et al. 2020). Accordingly, it is conceivable that small changes in the GNE activity can lead to subtle alterations in the glycan structures of few substrates leading to aberrant signalling and pathology (Hinderlich et al. 2004; Salama et al. 2005; Sela et al. 2020).

In our present study, we generated a new *Gne*-knockout model, based on the murine skeletal muscle cell line C2C12. We treated C2C12 *Gne*-knockout cells with the metabolites ManNAc, Ac_4_ManNAc, and Neu5Ac, respectively, to investigate their ability to restore (poly-)sialylation.

We selected two C2C12 *Gne*-knockout clones, which both lacked sialylation and were unable to differentiate into mature myotubes.

We further observed major differences in the ability to restore polysialylation when comparing ManNAc treatment of human kidney cells (HEK-293) and murine myoblasts (C2C12), respectively. These results suggest tissue and/or species differences in sialic acid metabolism. While HEK-293 *GNE*-knockout cells were able to use ManNAc as a substrate for sialic acid synthesis, this was not possible for the murine skeletal muscle *Gne*-knockout cell lines C2C12 and Sol8.

While a recent study implies, that exogenous ManNAc is phosphorylated by NAGK (Gorenflos Lopez et al. 2023), our results show that even with *Nagk* being expressed in C2C12 Gne-KO cells, there is no ManNAc phosphorylation. Thus, we suggest that further work is needed to identify the players that are involved in ManNAc metabolism in the absence of GNE.

High polarity of ManNAc impedes its diffusion across the plasma membrane resulting in poor cellular uptake whileperacetylation of ManNAc (Ac_4_ManNAc) increases it’s lipophilicity and allows administration of μM rather than mM concentrations (Agatemor et al. 2019). However, even Ac_4_ManNAc was unable to restore the level of polysialylation in C2C12 *Gne*-knockout cells. This could indicate, that the failure of processing ManNAc to Sia is due to the lack of suitable enzymes rather than a lack of permeability.

Additionally, it is known that both free and glycan-bound sialic acids can be endocytosed back into cells when needed, allowing lysosomal processes to recycle them and make them available again for glycan synthesis (see Fig. 1, part 4) (Verheijen et al. 1999; Huang et al. 2015). Intriguingly, high doses of sialic acid administered for 8 days in C2C12-*Gne*-knockout cells showed an adaption of sialylation and polysialylation to C2C12 wild type levels. Sialic acid treatment was also able to rescue the differentiation-deficient phenotype in clone 26 but not in clone 24. The differences between the two *Gne*-knockout clones regarding sialic acid responsiveness and cell morphology could be due to single cell heterogeneity, consistent with the differences in NCAM and β-tubulin expression in both clones shown in Fig. 3. Notably, high doses of sialic acid appear to have a rather negative impact on wild type cell differentiation, as evidenced by impaired myotube formation and decreased MYH expression. These results suggest a tightly regulated fine-tuning of the sialic acid content of muscle cells.

The findings in this study are fundamental, showing the direct effects of Gne depletion on murine skeletal muscle cells. It is however of great interest, that our basic model shows good similarity with the patient-derived iPSC model of Schmitt and co-workers (Schmitt et al. 2022), providing a low-cost alternative to investigate the role of sialic acids in a skeletal muscular context. The results cannot be directly translated for GNEM patients but might help in a broader understanding of this rare genetic disease. In summary, we believe that further work is needed to fully understand and exploit the therapeutic potential of sialic acid and its precursors in the context of GNEM.

## Materials and methods

### Cell culture

C2C12, Sol8, and HEK-293 cells were cultured in DMEM (Dulbecco’s Modified Eagle’s Medium; 11,960,044; Gibco/Thermo Fisher Scientific; Waltham, MA, USA) supplemented with 10% FBS (Fetal Bovine Serum; A5256801; Gibco/Thermo Fisher Scientific; Waltham, MA, USA), 1% penicillin–streptomycin (P/S; 10,000 units/mL (P) and 10,000 μg/mL (S); 15,140,122; Gibco/Thermo Fisher Scientific; Waltham, MA, USA), and 1% L-glutamine (L-Gln; 200 mM; A2916801; Gibco/Thermo Fisher Scientific; Waltham, MA, USA) at 37 °C in a humidified atmosphere with 5% CO_2_. For C2C12 differentiation, normal growth medium (GM) was replaced by differentiation medium (DM). This medium consists of DMEM supplemented with 2% horse serum (S-30-L; c.c.pro GmbH; Oberdorla, Germany), 1% P/S, and 1%L-Gln. HEK-293 (ACC 305) and C2C12 cells (ACC 565) were purchased from DSMZ (Braunschweig, Germany). Sol8 wild type and Sol8 *Gne*-knockout cells were kindly provided by Stella Mitrani-Rosenbaum (Hadassah–The Hebrew University Medical Center, Israel) (Ilouz et al. 2022). Peracetylated *N-*acetylmannosamine (Ac_4_-ManNAc) was purchased from Synvenio (SV3881; Nijmegen, The Netherlands), *N*-acetylmannosamine (ManNAc) was from New Zealand Pharmaceuticals Limited (Palmerston North, New Zealand), and *N*-acetylneuraminic acid (Neu5Ac) was from Molekula Group GmbH (39596039-10 g; Munich, Germany).

### CRISPR/Cas9 gene editing

Commercial CRISPR/Cas9 *Gne*-knockout and HDR plasmids were purchased from Santa Cruz Biotechnology (sc-424509-HDR). Briefly, 25,000 C2C12 wild type cells were seeded in full growth medium without penicillin–streptomycin in a 6-well dish 24 h prior to transfection. Co-transfection of the CRISPR-KO and HDR-plasmids were done using UltraCruz® Transfection Reagent (sc-395739). Positive clones were selected via puromycin treatment (1.5 μg/mL) and single cell suspension was seeded into 96-well plates to obtain single cell clones. Peters et al. described the generation of the HEK-293 *GNE*-knockout cells, which is very similar to the generation of the C2C12 *Gne*-knockout cells (Peters et al. 2023).

### Western blot/immunoblot

For protein isolation, cells were washed with ice-cold PBS and harvested from the culture dish using a cell scraper to avoid membrane protein modifications. The cell pellet was lysed in RIPA buffer (25 mM Tris–HCl pH 7.6, 150 mM NaCl, 1% NP-40, 1 mM EDTA) containing 1x protease inhibitor cocktail (Sigma Aldrich; St. Louis, MO, USA), 1 mM NaVO_4_, and 1 mM PMSF. Following 30 min incubation on ice, total protein was isolated by centrifugation at 14,000 × g at 4 °C for 5 min and quantified using the Pierce™ BCA Protein Assay Kit (23,225; Thermo Fisher Scientific; Waltham, MA, USA). Equal amounts of protein were mixed with 5 × SDS-laemmli buffer (containing 50 mM DTT) and separated on a 10% gel. Proteins were transferred on a nitrocellulose-membrane and stained with Ponceau S as loading control. Membranes were blocked with 5% skimmed milk in TBS-Tween (TBS-T) for 1 h at room temperature prior to incubation with primary antibody over night at 4 °C with agitation. On the next day, membranes were washed with TBS-T and subsequently incubated with secondary antibody for 1 h at room temperature (Goat anti-mouse IgG H&L HRP, 1:10,000, ab6789; abcam; Cambridge, UK). After three repeated washing steps with TBS-T, Immobilon® Forte Western HRP Substrate was used for detection (Merck, Germany) using the ChemiDoc MP imaging system from Bio-Rad Laboratories (Hercules, CA, USA). Signal quantification was performed using ImageJ Software.

The following primary antibodies were used:

**Table:** 

name	target protein/modification	dilution	company	ordering number
GLCNE (H-10)	GNE	1:1000	Santa Cruz Biotechnology	sc-376,057
MYH (B-5)	Myosin heavy chains (MYH)	1:1000	Santa Cruz Biotechnology	sc-376,157
β-Tubulin (BT7R)	β-Tubulin	1:5000	Thermo Fisher Scientific	MA5–16308
GlcNAc kinase (G-5)	GlcNAc kinase (NAGK)	1:1000	Santa Cruz Biotechnology	sc-390499
PolySia 735	Polysialylation	1:1000	Kind gift from Rita Gerardy-Schahn (Hannover, Germany)	
NCAM 5B8	Neural cell adhesion molecule (NCAM)	1:1000	made in-house	

### qRT-PCR

Total RNA was isolated using the Quick-RNA Miniprep Kit (R1054; Zymo Research; Irvine, CA, USA). cDNA was synthesized, using 2 μg of total RNA and SuperScript™ II reverse transcriptase (18,064,022; 2,000 units; Thermo Fisher Scientific; Waltham, MA, USA), following the manufacturer’s instructions. RT-qPCR was performed using qPCR SybrMaster (PCR-372S; Jena Bioscience; Jena, Germany) and the CFX Connect™ Real-Time PCR Detection System (1,855,201; Bio-Rad; Hercules, CA, USA). Cq values were normalized to the housekeeping gene *Gapdh*, and relative gene expression was calculated using the ΔΔCq-method. Primer sequences used:

**Table:** 

**gene name**	**direction**	**sequence**
*Gapdh*	forward	CCTGGAGAAACCTGCCAAGTATG
*Gapdh*	reverse	AGAGTGGGAGTTGCTGTTGAAGTC
*St8sia2*	forward	GTGTGGAGTGGGTCAATGCT
*St8sia2*	reverse	TCAATGCCCCCTGTTCATGT
*St8sia4*	forward	GCTGGGACAACCAGGACTTT
*St8sia4*	reverse	ACGTCACGTTCCGCATCTAA
*Nagk*	forward	GGTAGTATGGCCGCGCTTTA
*Nagk*	reverse	GGTGTGCAATCCAGTAGGCT

### Resorcinol assay (quantification of sialic acids)

Total amounts of sialic acids were quantified using the periodate-resorcinol assay. Briefly, cells were washed three times with ice-cold PBS and lysed in 250 μl PBS via freeze–thaw cycles in liquid nitrogen. All samples and the standard curve were oxidised with 5 μl of 0.4 M periodic acid for >10 min on ice. Then, 500 μl of the following solution were added to each sample and mixed by vortexing: 0.6% resorcinol, 0.25 mM CuSO_4_, 36% H_2_0, 44% concentrated HCl. Samples were incubated at 100 °C for exactly 15 min and allowed to cool down to room temperature afterwards, before adding 500 μl of *tert*-butanol. To remove any particular remnants, the samples were briefly centrifuged. OD_630_ was measured in triplicate in a 96-well plate and sialic acid levels were calculated from the standard curve and normalized to the total protein amount of each sample.

### Statistical analysis

Student’s *t*-tests were used to calculate *P*-values for statistical analysis of results.

## Supplementary Material

Supplementary_Data_cwae004

## Data Availability

The authors confirm that the data supporting the findings of this study are available within the article [and/or] its supplementary materials. Raw data were generated at the Martin Luther University Halle-Wittenberg. Derived data supporting the findings of this study are available from the corresponding author [CN] on request.

## References

[ref1] Agatemor C, Buettner MJ, Ariss R, Muthiah K, Saeui CT, Yarema KJ. Exploiting metabolic glycoengineering to advance healthcare. Nat Rev Chem. 2019:3(10):605–620. 31777760 10.1038/s41570-019-0126-y PMC6880190

[ref2] Allen MB, Walker DG. Kinetic characterization of n-acetyl-d-glucosamine kinase from rat liver and kidney. Biochem J. 1980:185(3):577–582. 6248026 10.1042/bj1850577 PMC1161433

[ref3] Argov Z, Yarom R. "Rimmed vacuole myopathy" sparing the quadriceps. A unique disorder in iranian jews. J Neurol Sci. 1984:64(1):33–43. 6737002 10.1016/0022-510x(84)90053-4

[ref4] Bhide GP, Colley KJ. Sialylation of n-glycans: mechanism, cellular compartmentalization and function. Histochem Cell Biol. 2017:147(2):149–174. 27975143 10.1007/s00418-016-1520-x PMC7088086

[ref5] Blazev R, Ashwood C, Abrahams JL, Chung LH, Francis D, Yang P, Watt KI, Qian H, Quaife-Ryan GA, Hudson JE, et al. Integrated glycoproteomics identifies a role of n-glycosylation and galectin-1 on myogenesis and muscle development. Mol Cell Proteomics. 2021:20:100030. 33583770 10.1074/mcp.RA120.002166 PMC8724610

[ref6] Carrillo N, Malicdan MC, Huizing M. GNE myopathy: Etiology, diagnosis, and therapeutic challenges. Neurotherapeutics. 2018:15(4):900–914. 30338442 10.1007/s13311-018-0671-y PMC6277305

[ref7] Devi S, Yadav R, Chanana P, Arya R. Fighting the cause of alzheimer's and GNE myopathy. Front Neurosci. 2018:12:669. 30374284 10.3389/fnins.2018.00669 PMC6196280

[ref8] Eisenberg I, Avidan N, Potikha T, Hochner H, Chen M, Olender T, Barash M, Shemesh M, Sadeh M, Grabov-Nardini G, et al. The udp-n-acetylglucosamine 2-epimerase/n-acetylmannosamine kinase gene is mutated in recessive hereditary inclusion body myopathy. Nat Genet. 2001:29(1):83–87. 11528398 10.1038/ng718

[ref9] Gorenflos Lopez JL, Schmieder P, Kemnitz-Hassanin K, Asikoglu HC, Celik A, Stieger CE, Fiedler D, Hinderlich S, Hackenberger CPR. Real-time monitoring of the sialic acid biosynthesis pathway by nmr. Chem Sci. 2023:14(13):3482–3492. 37006695 10.1039/d2sc06986e PMC10055903

[ref10] Hinderlich S, Stasche R, Zeitler R, Reutter W. A bifunctional enzyme catalyzes the first two steps in n-acetylneuraminic acid biosynthesis of rat liver. Purification and characterization of udp-n-acetylglucosamine 2-epimerase/n-acetylmannosamine kinase. J Biol Chem. 1997:272(39):24313–24318. 9305887 10.1074/jbc.272.39.24313

[ref11] Hinderlich S, Berger M, Keppler OT, Pawlita M, Reutter W. Biosynthesis of n-acetylneuraminic acid in cells lacking udp-n-acetylglucosamine 2-epimerase/n-acetylmannosamine kinase. Biol Chem. 2001:382(2):291–297. 11308027 10.1515/BC.2001.036

[ref12] Hinderlich S, Salama I, Eisenberg I, Potikha T, Mantey LR, Yarema KJ, Horstkorte R, Argov Z, Sadeh M, Reutter W, et al. The homozygous m712t mutation of udp-n-acetylglucosamine 2-epimerase/n-acetylmannosamine kinase results in reduced enzyme activities but not in altered overall cellular sialylation in hereditary inclusion body myopathy. FEBS Lett. 2004:566(1–3):105–109. 15147877 10.1016/j.febslet.2004.04.013

[ref13] Huang C, Seino J, Wang L, Haga Y, Suzuki T. Autophagy regulates the stability of sialin, a lysosomal sialic acid transporter. Biosci Biotechnol Biochem. 2015:79(4):553–557. 25494612 10.1080/09168451.2014.991682

[ref14] Ilouz N, Harazi A, Guttman M, Daya A, Ruppo S, Yakovlev L, Mitrani-Rosenbaum S. In vivo and in vitro genome editing to explore gne functions. Front Genome Ed. 2022:4:930110. 10.3389/fgeed.2022.930110. 36237634 PMC9552322

[ref15] Krause S, Schlotter-Weigel B, Walter MC, Najmabadi H, Wiendl H, Muller-Hocker J, Muller-Felber W, Pongratz D, Lochmuller H. A novel homozygous missense mutation in the gne gene of a patient with quadriceps-sparing hereditary inclusion body myopathy associated with muscle inflammation. Neuromuscul Disord. 2003:13(10):830–834. 14678807 10.1016/s0960-8966(03)00140-8

[ref16] Lochmuller H, Behin A, Caraco Y, Lau H, Mirabella M, Tournev I, Tarnopolsky M, Pogoryelova O, Woods C, Lai A, et al. A phase 3 randomized study evaluating sialic acid extended-release for GNE myopathy. Neurology. 2019:92(18):e2109–e2117. 31036580 10.1212/WNL.0000000000006932 PMC6512882

[ref17] Malicdan MC, Noguchi S, Nonaka I, Hayashi YK, Nishino I. A gne knockout mouse expressing human gne d176v mutation develops features similar to distal myopathy with rimmed vacuoles or hereditary inclusion body myopathy. Hum Mol Genet. 2007:16(22):2669–2682. 17704511 10.1093/hmg/ddm220

[ref18] Malicdan MC, Noguchi S, Hayashi YK, Nonaka I, Nishino I. Prophylactic treatment with sialic acid metabolites precludes the development of the myopathic phenotype in the dmrv-hibm mouse model. Nat Med. 2009:15(6):690–695. 19448634 10.1038/nm.1956

[ref19] Maliekal P, Vertommen D, Delpierre G, Van Schaftingen E. Identification of the sequence encoding n-acetylneuraminate-9-phosphate phosphatase. Glycobiology. 2006:16(2):165–172. 16237198 10.1093/glycob/cwj050

[ref20] Milman, Krentsis I, Sela I, Eiges R, Blanchard V, Berger M, Becker Cohen M, Mitrani-Rosenbaum S. Gne is involved in the early development of skeletal and cardiac muscle. PLoS One. 2011:6(6):e21389. 10.1371/journal.pone.0021389. 21731727 PMC3123316

[ref21] Mitrani-Rosenbaum S, Yakovlev L, Becker Cohen M, Argov Z, Fellig Y, Harazi A. Pre clinical assessment of aavrh74.Mck.Gne viral vector therapeutic potential: robust activity despite lack of consistent animal model for GNE myopathy. J Neuromuscul Dis. 2022:9(1):179–192. 34806613 10.3233/JND-210755 PMC8842764

[ref22] Monzo HJ, Park TI, Dieriks BV, Jansson D, Faull RL, Dragunow M, Curtis MA. Insulin and igf1 modulate turnover of polysialylated neural cell adhesion molecule (psa-ncam) in a process involving specific extracellular matrix components. J Neurochem. 2013:126(6):758–770. 23844825 10.1111/jnc.12363

[ref23] Muhlenhoff M, Eckhardt M, Bethe A, Frosch M, Gerardy-Schahn R. Polysialylation of ncam by a single enzyme. Curr Biol. 1996:6(9):1188–1191. 8805371 10.1016/s0960-9822(02)70687-8

[ref24] Munster-Kuhnel AK, Tiralongo J, Krapp S, Weinhold B, Ritz-Sedlacek V, Jacob U, Gerardy-Schahn R. Structure and function of vertebrate cmp-sialic acid synthetases. Glycobiology. 2004:14(10):43R–51R. 10.1093/glycob/cwh113 15201214

[ref25] Niethamer TK, Yardeni T, Leoyklang P, Ciccone C, Astiz-Martinez A, Jacobs K, Dorward HM, Zerfas PM, Gahl WA, Huizing M. Oral monosaccharide therapies to reverse renal and muscle hyposialylation in a mouse model of GNE myopathy. Mol Genet Metab. 2012:107(4):748–755. 23122659 10.1016/j.ymgme.2012.10.011 PMC3504164

[ref26] Nonaka I, Sunohara N, Ishiura S, Satoyoshi E. Familial distal myopathy with rimmed vacuole and lamellar (myeloid) body formation. J Neurol Sci. 1981:51(1):141–155. 7252518 10.1016/0022-510x(81)90067-8

[ref27] Pertusati F, Morewood J. Synthesis of 2-acetamido-1,3,4-tri-o-acetyl-2-deoxy-d-mannopyranose −6-phosphate prodrugs as potential therapeutic agents. Curr Protoc. 2022:2(8):e500. 35976612 10.1002/cpz1.500 PMC12016457

[ref28] Peters E, Selke P, Bork K, Horstkorte R, Gesper A. Evaluation of n-acetylmannosamine administration to restore sialylation in gne-deficient human embryonal kidney cells. Front Biosci (Landmark Ed). 2023:28(11):300. 38062838 10.31083/j.fbl2811300

[ref29] Pogoryelova O, Gonzalez Coraspe JA, Nikolenko N, Lochmuller H, Roos A. GNE myopathy: from clinics and genetics to pathology and research strategies. Orphanet J Rare Dis. 2018:13(1):70. 29720219 10.1186/s13023-018-0802-x PMC5930817

[ref30] Revel-Vilk S, Shai E, Turro E, Jahshan N, Hi-Am E, Spectre G, Daum H, Kalish Y, Althaus K, Greinacher A, et al. Gne variants causing autosomal recessive macrothrombocytopenia without associated muscle wasting. Blood. 2018:132(17):1851–1854. 30171045 10.1182/blood-2018-04-845545 PMC6202914

[ref31] Roseman S, Jourdian GW, Watson D, Rood R. Enzymatic synthesis of sialic acid 9-phosphates. Proc Natl Acad Sci U S A. 1961:47(7):958–961. 13743311 10.1073/pnas.47.7.958 PMC221309

[ref32] Salama I, Hinderlich S, Shlomai Z, Eisenberg I, Krause S, Yarema K, Argov Z, Lochmuller H, Reutter W, Dabby R, et al. No overall hyposialylation in hereditary inclusion body myopathy myoblasts carrying the homozygous m712t gne mutation. Biochem Biophys Res Commun. 2005:328(1):221–226. 15670773 10.1016/j.bbrc.2004.12.157

[ref33] Schmitt RE, DYT S, Cho DS, Kirkeby LA, Resch ZT, Liewluck T, Niu Z, Milone M, Doles JD. Myogenesis defects in a patient-derived ipsc model of hereditary GNE myopathy. NPJ Regen Med. 2022:7(1):48. 36085325 10.1038/s41536-022-00238-3 PMC9463157

[ref34] Schwarzkopf M, Knobeloch KP, Rohde E, Hinderlich S, Wiechens N, Lucka L, Horak I, Reutter W, Horstkorte R. Sialylation is essential for early development in mice. Proc Natl Acad Sci U S A. 2002:99(8):5267–5270. 11929971 10.1073/pnas.072066199 PMC122758

[ref35] Sela I, Goss V, Becker-Cohen M, Dell A, Haslam SM, Mitrani-Rosenbaum S. The glycomic sialylation profile of GNE myopathy muscle cells does not point to consistent hyposialylation of individual glycoconjugates. Neuromuscul Disord. 2020:30(8):621–630. 32736841 10.1016/j.nmd.2020.05.008

[ref36] Seppala R, Tietze F, Krasnewich D, Weiss P, Ashwell G, Barsh G, Thomas GH, Packman S, Gahl WA. Sialic acid metabolism in sialuria fibroblasts. J Biol Chem. 1991:266(12):7456–7461. 2019577

[ref37] Seppala R, Lehto VP, Gahl WA. Mutations in the human udp-n-acetylglucosamine 2-epimerase gene define the disease sialuria and the allosteric site of the enzyme. Am J Hum Genet. 1999:64(6):1563–1569. 10330343 10.1086/302411 PMC1377899

[ref38] Varki A . Sialic acids in human health and disease. Trends Mol Med. 2008:14(8):351–360. 18606570 10.1016/j.molmed.2008.06.002 PMC2553044

[ref39] Verheijen FW, Verbeek E, Aula N, Beerens CE, Havelaar AC, Joosse M, Peltonen L, Aula P, Galjaard H, van der Spek PJ, et al. A new gene, encoding an anion transporter, is mutated in sialic acid storage diseases. Nat Genet. 1999:23(4):462–465. 10581036 10.1038/70585

[ref40] Xu X, Wang AQ, Latham LL, Celeste F, Ciccone C, Malicdan MC, Goldspiel B, Terse P, Cradock J, Yang N, et al. Safety, pharmacokinetics and sialic acid production after oral administration of n-acetylmannosamine (mannac) to subjects with GNE myopathy. Mol Genet Metab. 2017:122(1–2):126–134. 28641925 10.1016/j.ymgme.2017.04.010 PMC5949875

[ref41] Yabe I, Higashi T, Kikuchi S, Sasaki H, Fukazawa T, Yoshida K, Tashiro K. Gne mutations causing distal myopathy with rimmed vacuoles with inflammation. Neurology. 2003:61(3):384–386. 12913203 10.1212/01.wnl.0000061520.63546.8f

[ref42] Yoshioka W, Nishino I, Noguchi S. Recent advances in establishing a cure for GNE myopathy. Curr Opin Neurol. 2022:35(5):629–636. 35959526 10.1097/WCO.0000000000001090

