# Supplementary material for: GNE deficiency impairs Myogenesis in C2C12 cells and cannot be rescued by ManNAc supplementation
Source: Glycobiology. 2024 Jan 15;34(3):cwae004. doi: 10.1093/glycob/cwae004 (PMC10987290; doi:10.1093/glycob/cwae004)
Supplement: Supplementary_Data_cwae004 [file supplementary_data_cwae004.docx]

**Supplements**


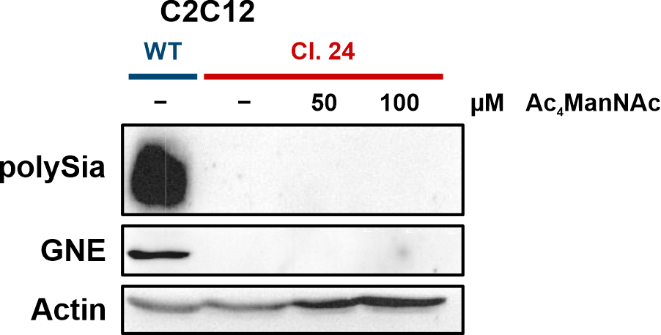


Suppl. Fig. 1: **Treatment of the C2C12 Gne-knockout clone 24 with different concentrations of Ac_4_ManNAc. This is one representative Western blot of three replicates.**


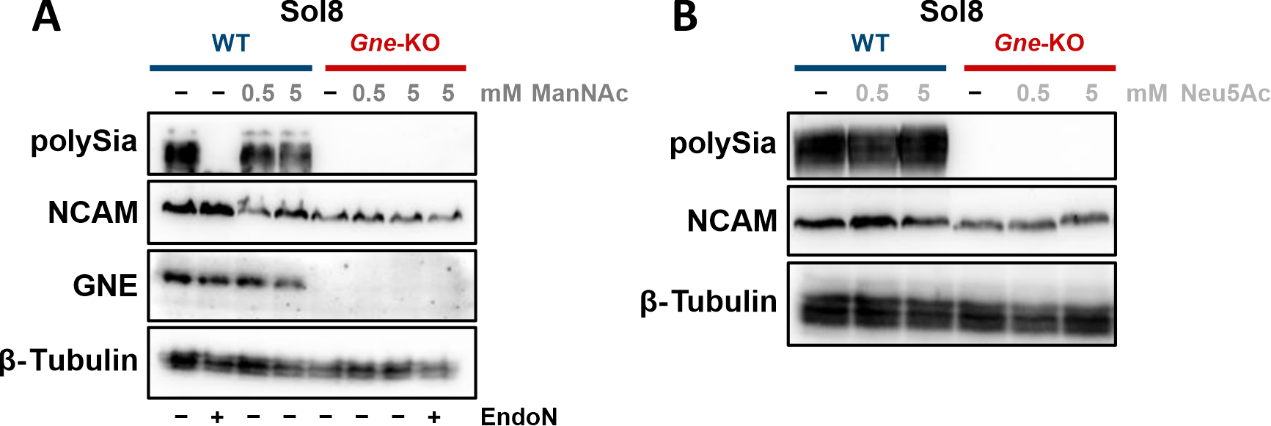


Suppl. Fig. 2: **Treatment of Sol8 wild type and Gne-knockout cells with ManNAc and Neu5Ac for 24 h. EndoN (Endoneuraminidase-N) hydrolyzes α2-8 linkages of polySia from NCAM as additional internal control.**


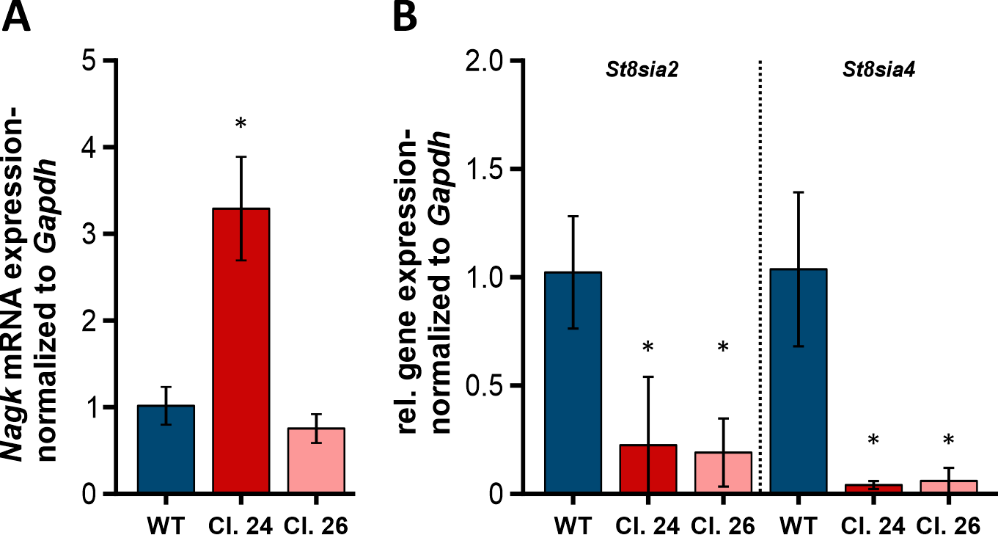


Suppl. Fig. 3: **qPCR analysis of the mRNA expression of A) Nagk and B) St8sia2 and St8sia4 normalized to the expression of Gapdh.**
